# Temperament, parenting, mental disorders, life stressors and help-seeking behavior of Asian adolescent suicide attempters: A case control study

**DOI:** 10.3389/fpsyt.2022.999089

**Published:** 2022-09-29

**Authors:** John Chee Meng Wong, Christel Lynne Chang, Liang Shen, Nyein Nyein, Adrian Seng Wei Loh, Natalie Huijing Yap, Leoniek Mirjam Kroneman, Lei Feng, Chay Hoon Tan

**Affiliations:** ^1^Department of Psychological Medicine, Yong Loo Lin School of Medicine, National University of Singapore, Singapore, Singapore; ^2^Department of Psychological Medicine, National University Health System, Singapore, Singapore; ^3^Biostatistics Unit, Yong Loo Lin School of Medicine, National University of Singapore, Singapore, Singapore; ^4^Healthy Longevity Translational Research Programme, Yong Loo Lin School of Medicine, National University of Singapore, Singapore, Singapore; ^5^Centre for Healthy Longevity, National University Health System, Singapore, Singapore; ^6^Department of Pharmacology, Yong Loo Lin School of Medicine, National University of Singapore, Singapore, Singapore

**Keywords:** adolescent, risk factor (RF), suicide attempt (SA), temperament, parenting, mental disorder (disease), life stressor, help-seeking

## Abstract

**Purpose:**

The need to elucidate risk factors for adolescent suicide is urgent, as suicide consistently ranks among the top causes of death globally. Understanding suicide risk factors could inform more effective interventions. Previous studies have identified certain risk factors associated with suicide, but there is a paucity of research among adolescent and multi-ethnic Asian populations.

**Materials and methods:**

This case-control study sampled 13-to-19-year-old Asian adolescents who had attempted suicide (*N* = 60) and controls (*N* = 58) matched by age, ethnicity and gender at group-level (73.7% female). Life stressors, temperament, parenting style, mental health conditions and help-seeking behavior were examined.

**Results:**

All domains of life stress apart from emerging adult responsibility were higher among cases than controls, especially home life, peer pressure and romantic relationships. Suicide attempters tended to avoid new situations, be less adaptable to changes, have a negative outlook and irregular sleep-wake cycle. Additionally, they perceived their parents to be significantly more aggressive, neglecting, rejecting and cold, while parents’ perceptions of their own parenting were only significantly different in the domain of parental neglect. Cases were more likely to exhibit disorders of disruptive behavior, eating, mood, anxiety, symptoms of schizophrenia and experience of disturbing events. Significant differences were also found for 10 out of 12 Axis II disorders, particularly borderline, depressive, and avoidant personality disorder traits. No significant case-control differences were found regarding overall rates of help-seeking.

**Conclusion:**

Findings from this study may help in suicide prevention efforts through more tailored interventions.

## Introduction

Suicide is consistently ranked among the top causes of adolescent death globally (1). Early identification of at-risk adolescents is crucial, as suicide often does not happen on impulse, but results from interacting individual, social, and environmental factors. By far the greatest risk factor for completed suicide is a previous suicide attempt (1, 2). By discerning risk factors associated with suicide attempt, early identification of those at risk could help prevent future attempted and completed suicides through more tailored interventions. Moreover, suicidality is often under-reported in national statistics through completed suicides being incorrectly classified as accidents (3). Early identification of suicide risk is thus additionally pertinent in order to aid in post-mortem investigations to more accurately distinguish suicide cases (4).

A broad range of external risk factors for suicidal outcomes has previously been found. For instance, life stressors associated with adolescent suicidality may include relationship issues and financial strain (5). Perceived parenting style may also influence adolescent suicide risk. Research in other populations has found rejecting-neglecting parenting and low levels of care to be associated with suicidal behavior (6, 7). Further examining these factors in Asian adolescents is necessary to elucidate risk factors for this under-studied population.

As per the diathesis-stress model (8), the impact of environmental stressors may be compounded by biological vulnerabilities, such as individual temperament or mental disorders. Maser et al. (9) found that completion of suicide within 12 months was predicted by clinical variables such as substance use or anxiety, while completion of suicide beyond 12 months was better predicted by temperamental factors such as impulsivity. Other characteristics previously found to be associated with suicide attempt include negative emotionality and neuroticism (10, 11). However, there are limited studies examining temperamental risk factors in adolescents. A consideration of temperament’s association with suicidal outcomes is necessary, as temperament and psychopathology share a common neurochemical basis (12, 13).

Regarding mental disorders, about 90% of individuals who committed suicide had a previously identifiable psychiatric disorder (14), although this figure varies with geographic location and ethnicity. Lower prevalence of psychiatric disorders among Asian populations has been found, although this might reflect social stigma around mental illness, thus reducing help-seeking behavior and rate of reporting (15). Seeking help from others may support the learning of effective social skills and problem-solving strategies, which could facilitate adolescents’ ability to weather stressful life events. It may also reflect the presence of a supportive social environment, which has been found to be associated with positive psychosocial outcomes (16). Failure to seek help from appropriate channels could lead to suicide completion (17).

The present study investigated the life stressors, temperament, parenting experienced, mental disorders and help-seeking behavior of Asian adolescents with history of suicide attempt in an exploratory manner. The hypotheses were that certain life stressors, temperament profiles, parenting experienced and mental disorders would be associated with attempt of suicide. To clarify these relationships, the strength of these associations were examined to provide insight into the specific factors that more strongly increase the risk of suicidal behavior. All variables examined were adolescent-reported, with the addition of parent-reported parenting style. A consideration of the parental perspective is pertinent since such contextual variables are valuable to account for in taxonomies of mental disorders and temperament (18).

As Singapore is a multi-ethnic Asian society (19), sampling participants from this pool could enable a generalizable study of Asian adolescents, not confined to a particular ethnicity. Findings could form a broad foundation for future studies examining the implementation of suicide prevention strategies in Asian populations. To our knowledge, this is the first study to explore this set of suicide-attempt risk factors in adolescents of different ethnicities from a single Asian country. As data collection for this study was carried out prior to the COVID-19 pandemic, the present sample of adolescents were not subject to the pandemic-related lifestyle or behavioral stressors that may further exacerbate suicide risk (20).

## Materials and methods

### Participants

In this case-control study, 13-to-19-year-old adolescents and their parents or primary caregivers were recruited sequentially as they presented to National University Hospital’s Emergency Medicine Department or associated clinical or community services during the study period. Cases were adolescents (*N* = 61) who had attempted suicide, while controls (*N* = 63) were adolescents who presented with acute medical conditions without severe morbidity, matched to cases by age, ethnicity and gender at group level. 68 and 71 parents or main caregivers were recruited for cases (27 fathers, 40 mothers) and controls (19 fathers, 48 mothers) respectively. One case was excluded as they did not meet eligibility criteria. Five controls were excluded due to inability to contact (*N* = 1) or documented past suicide attempt or self-harm (N = 4). The final sample consisted of 252 participants (60 cases, 67 parents; 58 controls, 67 parents).

Adolescents’ average age was 16.2-years-old (*sd* = 1.85; range = 13–19). 73.7% were female. Majority were of Chinese ethnicity (50.8%), followed by Indian (24.6%), Malay (20.3%) and others (4.3%). Participants identified their religious affiliation as Muslim (26.3%), Hindu/Sikh (21.1%), Christian (18.6%), no religion (18.6%) and Buddhist/Taoist (15.2%). 71.2% of the sample attended secondary school, 28.0% attended post-secondary school, and one adolescent was in primary school. There were no significant case-control differences in any demographic measure. An outline of demographic characteristics broken down according to participant groups (cases and controls) is given in Table 1.

**TABLE 1:** Demographic characteristics of adolescents.

		Suicide attempters (*N* = 60)	Control (*N* = 58)
Age	13 to 16 years	29 (48.3%)	36 (62.1%)
	17 to 19 years	31 (51.7%)	22 (37.9%)
Gender	Male	17 (28.3%)	14 (24.1%)
	Female	43 (71.7%)	44 (75.9%)
Ethnicity	Chinese	29 (48.3%)	31 (54.2%)
	Malay	13 (21.7%)	11 (18.6%)
	Indian	15 (25%)	14 (23.7%)
	Others	3 (5.1%)	2 (3.5%)
Religion	Buddhist/Taoist	6 (10%)	12 (20.7%)
	Muslim	16 (26.7%)	15 (25.9%)
	Christian	12 (20%)	10 (17.2%)
	Hindu/Sikh	12 (20%)	13 (22.4%)
	No Religion	14 (23.3%)	8 (13.8%)
Education	Secondary and below	43 (71.7%)	41 (70.7%)
	Technical education (ITE)	3 (5%)	5 (8.6%)
	Pre-university/Polytechnic	13 (21.7%)	12 (20.7%)
Housing type	1-2 room HDB (public apartment)	4 (6.7%)	1 (1.7%)
	3 room HDB (public apartment)	11 (18.3%)	8 (13.6%)
	4-5 room HDB/Executive (public apartment)	40 (66.6%)	35 (60.3%)
	Private apartment/Condominium	3 (5%)	9 (15.5%)
	Landed Housing	2 (3.3%)	5 (8.6%)
Housing ownership	Owner-occupied	55 (91.7%)	54 (91.5%)
	Rental	5 (8.3%)	5 (8.5%)
No. of People staying with adolescent	1 to 5	54 (90%)	49 (83.1%)
	6 to 9	6 (10%)	10 (16.9%)

Ethical approval for this project had been obtained from the Domain-Specific Review Board of the National Healthcare Group.

### Measures

To differentiate suicidal intent of the adolescents in the case and control groups, the Columbia-Suicide Severity Rating Scale (C-SSRS) (21) was used, which measures suicidal ideation, its severity, and suicidal behavior. The Adolescent Stress Questionnaire (ASQ) (22) was used to profile domains of adolescent stress resulting from various life stressors (α = 0.96). The present study utilized the Dimensions of Temperament Survey-Revised (DOTS-R) (23) to profile dimensions of adolescent temperament (α = 0.62 to 0.89). Parenting style was assessed via the Parental Control Scale (PCS) (24) and Parental Acceptance-Rejection Questionnaires (PARQ) (25) that were administered to both adolescents and their parents. The PCS (α = 0.73) assesses the perceptions of parental behavioral control as experienced by the adolescent or administered by the adult themselves, while the PARQ (α = 0.89) examines the parent-adolescent relationship in terms of dimensions of acceptance and rejection. Axis I mental disorders were examined using the Youth’s Inventory-4 Self Report (YI-4) (26), which evaluates emotional and behavioral disorders based on the Diagnostic and Statistical Manual-IV (DSM-IV) diagnostic criteria (α = 0.97). Axis II disorders were chiefly assessed via the Personality Diagnostic Questionnaire–4 (PDQ-4) (27), which examines 12 personality disorder traits (PDTs) from the DSM-IV (α = 0.94). Lastly, a self-constructed questionnaire comprising three questions was administered to evaluate *adolescents’* help-seeking behavior. Further details of all measurement instruments are displayed in Table 2.

**TABLE 2:** Details of measurement instruments.

Measurement instrument	Purpose	Details
Columbia-Suicide Severity Rating Scale	Screening tool to distinguish suicidal intent in adolescents	Semi-structured interview
Adolescent Stress Questionnaire	To profile adolescents’ life stressors	54-item self-report 5-point Likert scale 10 dimensions: future uncertainty, school performance, peer pressure, school-leisure conflict, home life, school attendance, financial pressure, romantic relationships, teacher interaction and emerging adult responsibility
Dimensions of Temperament Survey-Revised	To profile adolescents’ temperament	54-item self-report 4-point Likert scale 9 dimensions: activity level-general, activity level-sleep, task orientation, rhythmicity-daily habits, rhythmicity-eating, rhythmicity-sleep, approach-withdrawal, flexibility-rigidity and mood
Parental Control Scale	To profile the parenting style experienced and quality of the parent-adolescent relationship, reported by both adolescents and caregivers	13-item self-report 4-point Likert scale
Parent Adolescent Relationship Questionnaire	To profile the parenting style experienced and quality of the parent-adolescent relationship, reported by both adolescents and caregivers	60-item self-report 4-point Likert scale 4 sub-scales: coldness/lack of affection, hostility/aggression, indifference/neglect, and undifferentiated rejection Undifferentiated rejection refers to the perception of one’s parent to be rejecting in a way that is not distinctly unaffectionate, aggressive or neglecting
Youth’s Inventory-4 Self Report	To profile the behavioral, cognitive, and affective symptoms in 12-to-18-year-old adolescents from 18 different Axis I and II disorders based on the DSM-IV	128-item self-report 4-point Likert scale: “never,” “sometimes,” “often,” or “very often” Responses of “often” or “very often” were counted and compared with the Symptom Criterion score (i.e., minimum number of symptoms required for a DSM-IV diagnosis), resulting in binary scores (0 = no symptoms, 1 = symptoms present)
Personality Diagnostic Questionnaire–4	To profile the 12 personality disorder traits in adolescents based on the DSM-IV	99-item self-report True/false questions Embedded “Too Good” and “Suspect Questionnaire” scales help to ensure the questionnaire’s validity by identifying under-reporting and lying or random responses, respectively
Self-constructed questionnaire	To profile the help-seeking avenues and behavior of adolescents	Adolescents were asked to indicate if and from whom they had sought help regarding their emotional distress or desire to be dead in the past 12 months Help-seeking avenues included: counselor/psychologist, general practitioner, friends, mother, father, siblings, relatives, teacher, internet, telephone, religion and colleagues

### Data analysis

Data analysis was conducted on the IBM SPSS Statistics version 25. Pearson Chi-square test or Fisher’s Exact test, whichever was more appropriate, was used to compare the categorical variables between case and control group, and p-value and odds ratio (OR) were reported. For some variables in which the cross table contained a cell with observed value zero, a modified Haldane-Anscombe (28) correction was applied, whereby 0.5 was added to each cell for calculation. Student’s *t*-test was conducted to compare the continuous variables between case and control groups, and p-value, mean difference with 95% CI were reported.

## Results and discussion

### Life stressors

**FIGURE 1:**
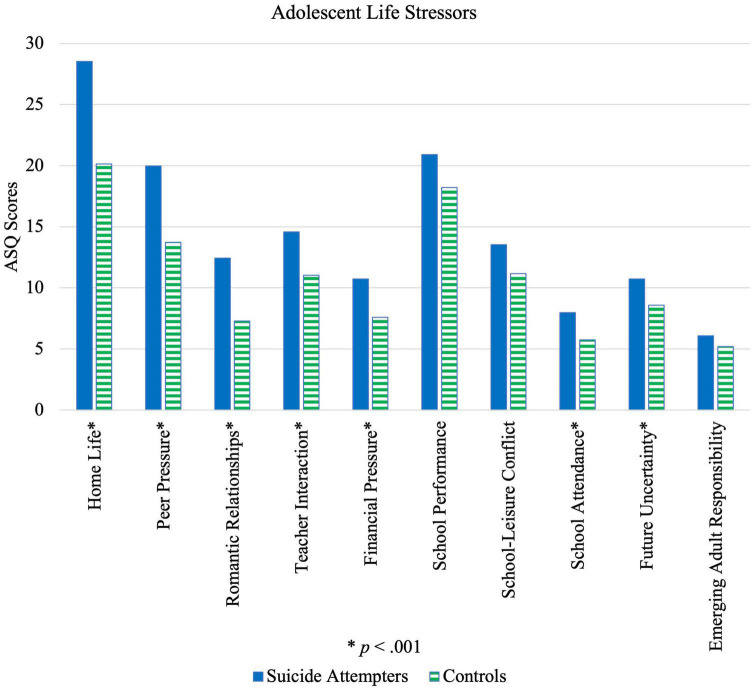
Illustration of differences in life stressors reported by suicide attempters and control group.

Across 13-to-19-year-old adolescents, cases had higher total stress levels compared to controls. Scores were significantly higher for suicide attempters than controls in all domains of the ASQ apart from emerging adult responsibility, as seen in Table 3 and illustrated by Figure 1. All factors were found to be statistically significant among females (*p* < 0.01, *M*_A–B_ = 2.28 – 7.93, 95% CI [0.56, 11.67]), except for emerging adult responsibility (*p* = 0.81). However, for male adolescents, home life (*p* = 0.005, *M*_A–B_ = 10.20, 95% CI [3.28, 17.12]), peer pressure (*p* = 0.005, *M*_A–B_ = 6.50, 95% CI [2.09, 10.91]), romantic relationships (*p* = 0.004, *M*_A–B_ = 6.35, 95% CI [2.16, 10.54]), financial pressure (*p* < 0.001, *M*_A–B_ = 5.50, 95% CI [2.66, 8.34]) and emerging adult responsibility (*p* = 0.004, *M*_A–B_ = 2.91, 95% CI [0.85, 4.97]) were significant risk factors.

**TABLE 3:** Comparison of life stressors between suicide attempters and control group.

	Suicide attempters^a^ (*N* = 60)	Control group^a^ (*N* = 58)	*P*-value	Mean difference	95% CI
ASQ (Total)**	145.40 ± 39.09	108.59 ± 30.56	<0.001	36.81	23.99 – 49.64
Home life**	28.53 ± 9.38	20.14 ± 8.52	<0.001	8.40	5.12 – 11.67
Peer pressure**	19.97 ± 7.18	13.71 ± 5.44	<0.001	6.26	3.93 – 8.59
Romantic relationships**	12.43 ± 6.36	7.28 ± 4.06	<0.001	5.16	3.20 – 7.11
Teacher interaction**	14.57 ± 6.57	11.02 ± 3.67	<0.001	3.55	1.60 – 5.50
Financial pressure**	10.72 ± 4.49	7.59 ± 3.47	<0.001	3.13	1.66 – 4.60
School performance*	20.92 ± 7.24	18.21 ± 5.31	0.023	2.71	0.39 – 5.03
School-leisure conflict*	13.52 ± 5.37	11.16 ± 4.58	0.012	2.36	0.54 – 4.18
School attendance**	7.97 ± 3.73	5.74 ± 2.65	<0.001	2.23	1.04 – 3.41
Future uncertainty**	10.72 ± 3.10	8.57 ± 2.95	<0.001	2.15	1.04 – 3.25
Emerging adult responsibility	6.07 ± 2.83	5.19 ± 2.24	0.065	0.88	0.05 – 1.81

^a^Mean ± Standard Deviation; *p < 0.05; **p < 0.001.

Stratification of the study population into two age groups of 13-to-16-year-olds (secondary school students) and 17-to-19-year-olds (post-secondary school students) provided insight into the differential impact of stressors, given their differences in school experiences and phase of social development. For 13-to-16-year-olds, significant differences across all factors (*p* < 0.01, *M*_*A–B*_ = 2.69 – 9.83, 95% CI [1.34, 14.04]) were established between suicide attempters and controls, except for that of emerging adult responsibility (*p* = 0.32). For 17-to-19-year-olds, only home life (*p* = 0.018, *M*_*A–B*_ = 6.55, 95% CI [1.15, 11.96]), romantic relationships (*p* = 0.012, *M*_*A–B*_ = 4.38, 95% CI [1.02, 7.73]) and peer pressure (*p* = 0.021, *M*_*A–B*_ = 4.43, 95% CI [0.70, 8.17]) were statistically significant at *p* < 0.05.

Overall, statistically significant case-control differences were greatest for interpersonal relationships, which is consistent with previous research reporting that chronic interpersonal stress results in increased suicidal ideation (29). High relational stress could additionally reflect a lack of socio-emotional support, which may render an individual more vulnerable, since social support has been found to be a protective factor against suicide (30). Peer pressure having the largest effect size (*d* = 0.98) in the overall sample, as well as in the female (*d* = 0.91) and 13-to-16-year-old age group (*d* = 1.24) is unsurprising, since social acceptance is important for adolescents, forming a large part of one’s identity development (31). Interestingly, emerging adult responsibility was the only stressor that did not significantly differ between cases and controls, even in the 17-to-19-year-old age group. A potential explanation might be that adolescents face the same stress *vis a vis* transitioning to adulthood, and the difference between suicide attempters and controls lies in their ability to cope. Findings for adolescent temperament, as discussed below, provides additional insight.

In the older 17-to-19-year-old group, romantic relationships (*d* = 0.74) as a stressor had the greatest effect size, indicating that romantic relationships may crucially influence the risk of suicide attempt in older adolescents. School performance and attendance as risk factors were significant only for younger adolescents and females. As such, when screening students at risk for suicide attempt, using school performance and attendance as tools for early identification might not be accurate for older adolescents and males. Furthermore, in male adolescents, financial pressure had the largest effect size (*d* = 1.48). Financial literacy training may be considered to provide adolescents with resources and instill hope through knowledge of the availability of options. Finally, in younger compared to older adolescents, significant case-control differences were found in more avenues of stress, indicating that risk factors for suicide attempt are more salient and thus might be easier to identify when adolescents are younger. This elevates the importance of early identification in suicide prevention efforts.

### Temperament

**FIGURE 2:**
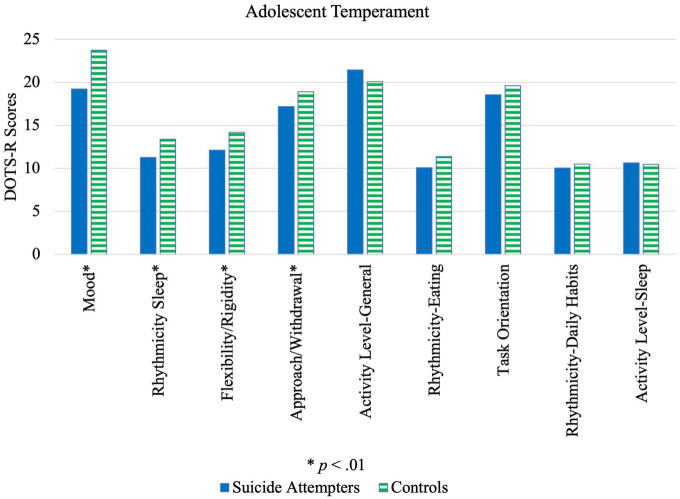
Illustration of differences in temperament characteristics between suicide attempters and control group.

As seen in Table 4 and illustrated by Figure 2, adolescents who attempted suicide tended to (a) avoid new persons/situations (approach/withdrawal); (b) be less adaptable to changes in routine/environment (flexibility/rigidity); (c) have a negative outlook (mood); and (d) have an irregular sleep-wake cycle (rhythmicity-sleep) significantly more than controls. Male adolescent cases had a significantly greater negative outlook (*p* = 0.003, *M*_A–B_ = –5.38, 95% CI [–8.82, 1.95]), irregular sleep-wake cycle (*p* = 0.009, *M*_A–B_ = –3.78, 95% CI [–6.53, 1.03]) and higher general activity levels (*p* = 0.04, *M*_A–B_ = 3.14, 95% CI [0.22, 6.06] than controls, while female cases tended to have a negative outlook (*p* < 0.001, *M*_A–B_ = –4.24, 95% CI [–6.46, 2.03]), avoid new persons/situations (*p* = 0.005, *M*_A–B_ = –2.27, 95% CI [–3.84, 0.71]) and be less adaptable to changes (*p* = 0.001, *M*_A–B_ = –2.07, 95% CI [–3.31, 0.83]).

**TABLE 4:** Comparison of temperament characteristics between suicide attempters and control group.

	Suicide attempters^a^ (*N* = 60)	Control group^a^ (*N* = 58)	*P*-value	Mean difference	95% CI
Mood**	19.23 ± 5.90	23.76 ± 3.94	< 0.001	–4.53	–6.36 to –2.69
Rhythmicity sleep*	11.28 ± 3.37	13.40 ± 4.30	0.004	–2.11	–3.52 to –0.71
Flexibility/Rigidity**	12.13 ± 2.73	14.19 ± 3.08	< 0.001	–2.06	–3.12 to –2.00
Approach/Withdrawal*	17.18 ± 3.76	18.91 ± 3.26	0.009	–1.73	–3.01 to –0.45
Activity level-general	21.45 ± 4.26	20.09 ± 4.81	0.106	1.36	–0.29 – 3.02
Rhythmicity-eating	10.07 ± 3.26	11.36 ± 4.05	0.058	–1.30	–2.63 – 0.04
Task orientation	18.55 ± 4.54	19.62 ± 3.78	0.167	–1.07	–2.60 – 0.45
Rhythmicity-daily habits	10.05 ± 2.63	10.50 ± 2.74	0.365	–0.45	–1.43 – 0.53
Activity level-sleep	10.65 ± 2.97	10.45 ± 3.37	0.731	0.20	–0.96 – 1.36

^a^Mean ± Standard Deviation; *p < 0.05, **p < 0.001.

Results from the present study corroborate with existing literature and extend their applicability to a multi-ethnic Asian population. For instance, previous studies found that adolescents who were more withdrawn in new social situations and less adaptable to changes were prone to developing depressive symptoms (32) and the tendency to express negative thoughts had been found associated with adolescent suicide attempt (33). Irregular sleep-wake patterns in adolescent suicide attempters have additionally been documented (34). Previously, Hurtig et al. (35) had reported that hyperactive and inattentive adolescents were more likely to have suicidal ideation compared to those who were not. In this sample, however, higher general activity levels were found for male but not female suicide attempters, compared to controls, although this was only significant at *p* < 0.05.

As a result of these tendencies toward withdrawal, inflexibility, low mood and irregular sleep patterns, adolescent suicide attempters may have less adaptive coping strategies when faced with similar life stressors as other adolescents. When compounded by greater intensity of life stressors, as previously discussed, their risk for suicidal behavior is amplified. Although, it is possible that these temperamental characteristics do not independently influence adolescents’ ability to cope but may reflect underlying mental disorders. Future research may investigate the combined or independent effects of these risk factors through regression analyses. Regardless, with these trait tendencies in mind, suicide prevention programs should be devised to support the development of effective coping strategies. The internalizing nature of many of these temperament traits may additionally cause these adolescents to fall under the radar. Implementing a regular check-in or buddy system may prove beneficial.

### Parenting style

**FIGURE 3:**
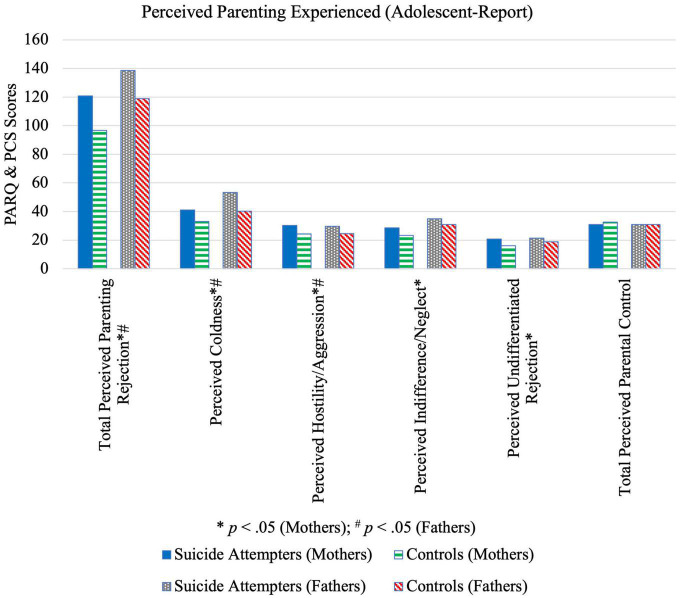
Illustration of differences in perceived parenting experienced reported by suicide attempters and control group adolescents.

From the adolescents’ perspective, findings indicate that mothers of adolescents who attempted suicide were significantly more aggressive, neglecting, rejecting, and cold, while fathers were perceived to be significantly more aggressive, rejecting and cold (Table 5 and Figure 3). Neither perceived maternal nor paternal control differed significantly between cases and controls. From the parents’ perspective, there were no significant case-control differences except for neglect (*p* = 0.044, *M*_A–B_ = 2.08, 95% CI [0.058, 4.09]) and maternal rejection (*p* = 0.042, *M*_A–B_ = 2.16, 95% CI [0.076, 4.25]).

**TABLE 5:** Comparison between suicide attempters and control group’s perceptions of their parents’ parenting.

Mothers’ parenting	Suicide attempters^a^ (*N* = 60)	Control group^a^ (*N* = 58)	*P*-value	Mean difference	95% CI
Total perceived parenting rejection**	120.73 ± 37.37	96.62 ± 25.85	<0.001	24.11	12.36 – 35.87
Perceived coldness**	41.00 ± 14.03	32.97 ± 10.02	<0.001	8.03	3.58 – 12.49
Perceived hostility/Aggression**	30.32 ± 10.20	24.33 ± 7.56	<0.001	5.99	2.70 – 9.28
Perceived indifference/Neglect*	28.62 ± 9.87	23.29 ± 6.87	0.001	5.32	2.21 – 8.43
Perceived undifferentiated rejection**	20.80 ± 7.64	16.03 ± 5.05	<0.001	4.77	2.40 – 7.14
Total perceived parental control	30.97 ± 7.58	32.54 ± 5.99	0.216	–1.58	–4.09 – 0.93

**Fathers’ parenting**	**Suicide attempters^a^** **(*N* = 58)**	**Control group^a^ (*N* = 56)**	***P*-value**	**Mean difference**	**95% CI**

Total perceived parenting rejection*	138.52 ± 35.71	118.96 ± 38.99	0.006	19.55	5.69 – 33.42
Perceived coldness**	53.29 ± 16.36	40.2 ± 13.66	<0.001	13.10	7.49 – 18.70
Perceived hostility/Aggression*	29.72 ± 11.52	24.61 ± 8.44	0.008	5.12	1.36 – 8.88
Perceived indifference/Neglect	34.86 ± 9.76	30.88 ± 12.07	0.054	3.99	–0.08 – 8.05
Perceived undifferentiated rejection	21.29 ± 8.40	18.93 ± 8.87	0.147	2.37	–0.84 – 5.57
Total perceived parental control	30.90 ± 10.23	30.91 ± 7.03	0.99	–0.01	–3.28 – 3.26

Total Perceived Parental Control was assessed *via* the Parental Control Scale. All other measures were derived from the Parent Adolescent Relationship Questionnaire. ^a^Mean ± Standard Deviation; *p < 0.05, **p < 0.001.

These findings are in line with results from the ASQ, which indicated large case-control differences in the dimension of home life. Previous studies have similarly found low levels of both maternal and paternal affection as perceived by adolescents who had attempted suicide (6). Additionally, the present study’s results for perceived parental hostility are consistent with Wagner and Cohen’s (36) finding that harsh maternal discipline was associated with adolescent suicide attempt. The present findings expand existing literature by adding that perceived *paternal* aggression is likewise higher among adolescent suicide attempters. Parental neglect and rejection had previously been found to increase the likelihood of suicide attempt (37). However, the present results indicate that only mothers’ but not fathers’ neglect was associated with attempted suicide.

With respect to parents’ perception of their own parenting style, surprisingly, no differences were found apart from that of neglect and maternal rejection. This could potentially be explained by (a) a lack of awareness regarding how their own behavior was perceived by their child, (b) modification of parenting practices following their child’s suicide attempt, or (c) social desirability bias resulting in inaccurate questionnaire responses. As such, when evaluating parenting styles, more weight should be given to adolescent- rather than parent-reports. Interventions must be developed to help parents modify their parenting styles.

### Mental health conditions

**FIGURE 4:**
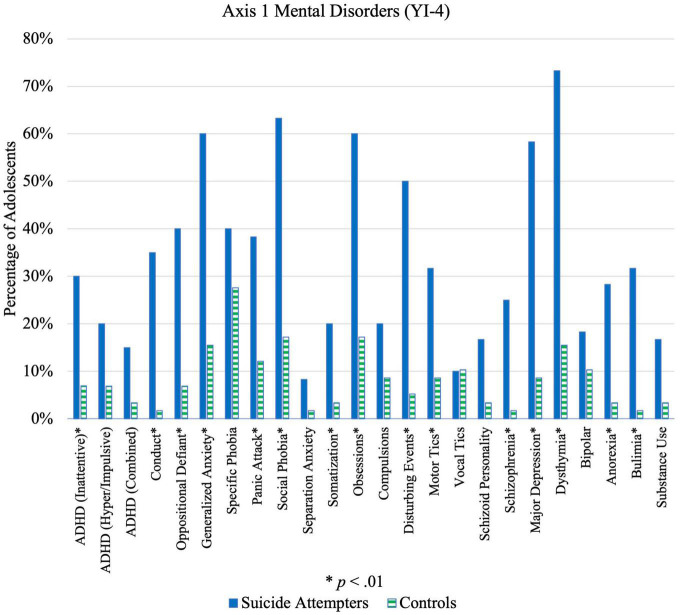
Illustration of differences in Axis 1 mental disorders between suicide attempters and control group.

For Axis I disorders, cases were more likely than controls to show symptoms of schizophrenia, eating disorders, disruptive behavior disorders, experience of disturbing events, mood disorders, and anxiety disorders, as shown in Table 6 (Figure 4). Cases were also more likely to show symptoms for more than two disorders; 90.0% of cases versus 41.4% of controls reported symptoms of at least two Axis I disorders (*p* < 0.001, OR = 12.75, 95%CI [4.73, 34.39]). The difference between cases and controls increased for possessing at least 3 Axis I disorder traits (85.0% cases, 27.6% controls; *p* < 0.001, OR = 14.88, 95% CI [5.97, 37.07]) and at least 4 Axis I disorder traits (80.0% cases, 20.7% controls; *p* < 0.001, OR = 15.33, 95% CI [6.26, 37.58]). Greater co-morbidity or co-occurrence of symptoms could thus be an independent risk factor for adolescent suicide attempt.

**TABLE 6:** Comparison of Axis I and II disorder traits between suicide attempters and control group.

	Sample size		*P*-value	Odds ratio	95% CI
	Suicide attempters^a^	Control^a^			
**YI-4**					
ADHD (Inattentive)*	18 (30.0%)	4 (6.92%)	0.001	5.79	1.82 – 18.38
ADHD (Hyper/Impulsive)*	12 (20%)	4 (6.9%)	0.038	3.38	1.02 – 11.17
ADHD (Combined)*	9 (15.0%)	2 (3.4%)	0.031	4.94	1.02 – 23.95
Conduct**	21 (35.0%)	1 (1.7%)	<0.001	30.69	3.96 – 237.71
Oppositional defiant**	24 (40.0%)	4 (6.9%)	<0.001	9.00	2.88 – 28.13
Generalized anxiety**	36 (60.0%)	9 (15.5%)	<0.001	8.17	3.39 – 19.66
Specific phobia	24 (40.0%)	16 (27.6%)	0.154	1.75	0.81 – 3.79
Panic attack*	23 (38.3%)	7 (12.1%)	0.001	4.52	1.76 – 11.66
Social phobia**	38 (63.3%)	10 (17.2%)	<0.001	8.29	3.51 – 19.60
Separation anxiety	5 (8.3%)	1 (1.7%)	0.1	5.18	0.59 – 45.78
Somatization*	12 (20.0%)	2 (3.4%)	0.005	7.00	1.49 – 32.84
Obsessions**	36 (60.0%)	10 (17.2%)	<0.001	7.20	3.06 – 16.93
Compulsions	12 (20%)	5 (8.6%)	0.078	2.65	0.87 – 8.07
Disturbing events**	30 (50.0%)	3 (5.2%)	<0.001	18.33	5.16 – 65.12
Motor tics*	19 (31.7%)	5 (8.6%)	0.002	4.91	1.69 – 14.27
Vocal tics	6 (10.0%)	6 (10.3%)	0.95	0.96	0.29 – 3.18
Schizoid personality*	10 (16.7%)	2 (3.4%)	0.018	5.60	1.17 – 26.79
Schizophrenia**	15 (25.0%)	1 (1.7%)	<0.001	19.00	2.42 – 149.32
Major depression**	35 (58.3%)	5 (8.6%)	<0.001	14.84	5.19 – 42.44
Dysthymia**	44 (73.3%)	9 (15.5%)	<0.001	14.97	6.01 – 37.29
Bipolar	11 (18.3%)	6 (10.3%)	0.217	1.95	0.67 – 5.66
Anorexia**	17 (28.3%)	2 (3.4%)	< 0.001	11.97	2.43 – 50.52
Bulimia**	19 (31.7%)	1 (1.7%)	<0.001	26.42	3.40 – 205.30
Substance use*	10 (16.7%)	2 (3.4%)	0.018	5.60	1.17 – 26.79
**PDQ-4**					
Antisocial**	17 (28.3%)	0 (0.0%)	<0.001	47.07^b^	2.75 – 804.32
Borderline**	53 (88.3%)	12 (20.7%)	<0.001	29.0	10.55 – 79.88
Depressive**	37 (61.7%)	6 (10.3%)	<0.001	13.9	5.17 – 37.61
Avoidant**	48 (80.0%)	18 (31.0%)	<0.001	8.89	3.83 – 20.64
Paranoid**	45 (75.0%)	18 (31.0%)	<0.001	6.67	2.98 – 14.94
Negativistic/Passive aggressive**	36 (60.0%)	13 (22.4%)	<0.001	5.19	2.32 – 11.61
Schizotypal**	32 (53.3%)	11 (19.0%)	<0.001	4.88	2.13 – 11.19
Dependent*	28 (46.7%)	10 (17.2%)	0.001	4.20	1.80 – 9.82
Obsessive-compulsive*	43 (71.7%)	29 (50%)	0.016	2.53	1.18 – 5.42
Schizoid*	28 (46.7%)	15 (25.9%)	0.02	2.51	1.15 – 5.45
Narcissistic	17 (28.3%)	11 (19.0%)	0.232	1.69	0.71 – 4.01
Histrionic	10 (16.7%)	7 (12.1%)	0.477	1.46	0.51 – 4.13
Too good scale	4 (6.7%)	9 (15.5%)	0.15	0.39	0.11 – 1.34
Suspect scale*	19 (31.7%)	6 (10.3%)	0.006	4.02	1.47 – 10.97

*P*-values calculated using Fishers’ Exact test. ^a^N(percentage); ^b^Modified Haldane-Anscombe correction applied due to presence of zero in contingency table, 0.5 was added to each cell; *p < 0.05, **p < 0.001.

**FIGURE 5:**
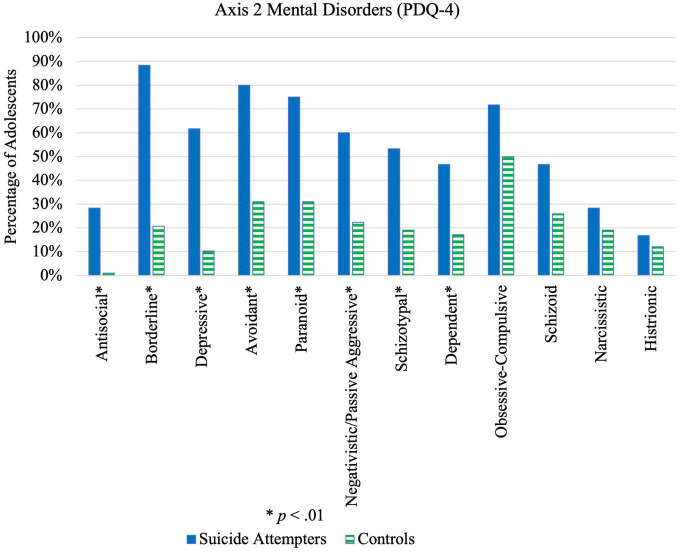
Illustration of differences in Axis 2 mental disorders between suicide attempters and control group.

Significant differences were also found between cases and controls for 10 out of 12 PDTs (Figure 5 and Table 6), particularly antisocial, borderline, depressive, and avoidant PDTs. Cases were also more likely to have a combination of PDTs compared to controls; 95.0% of cases versus 56.9% of controls possessed at least 2 PDTs (*p* < 0.001, OR = 14.39, 95% CI [4.03, 51.36]). A significant case-control difference was noted on the “Suspect” scale (Table 6), which suggests more cases were either lying, responding randomly, or not taking the questionnaire seriously compared to controls. No difference was found on the Too Good Scale, indicating that both cases and controls under-reported to the same extent.

Significant case-control differences for Axis I Disorders were consistent with findings from previous studies (38). Biological predispositions, combined with life stressors, place one at higher risk of developing psychiatric disorders (39). The higher intensity of stressful life events experienced by adolescent suicide attempters, as evidenced by their ASQ scores, could lead to increased vulnerability of developing mental disorders, which in turn leads to greater risk of suicidal outcomes (40). Furthermore, the higher proportion of comorbid Axis I disorder traits among cases compared to controls suggests that comorbidity, not just the presence of Axis I disorders, amplifies the risk of suicidality among adolescents and should be considered when identifying high-risk adolescents and developing suicide prevention strategies.

Results for PDTs among adolescent suicide attempters were similar to what had been found in a study by Brent et al. (41), where suicide attempters showed a greater number of symptoms associated with avoidant and borderline personality disorders compared to controls. Most existing studies primarily examined effects of borderline personality disorder on suicide attempt. The present findings highlight the importance of additionally considering other Axis II disorders when investigating risk factors. Importantly, histrionic PDTs differed insignificantly between cases and controls, highlighting the fact that adolescents who attempt suicide may not be simply attention-seeking but should be taken seriously.

### Help-seeking behavior

**FIGURE 6:**
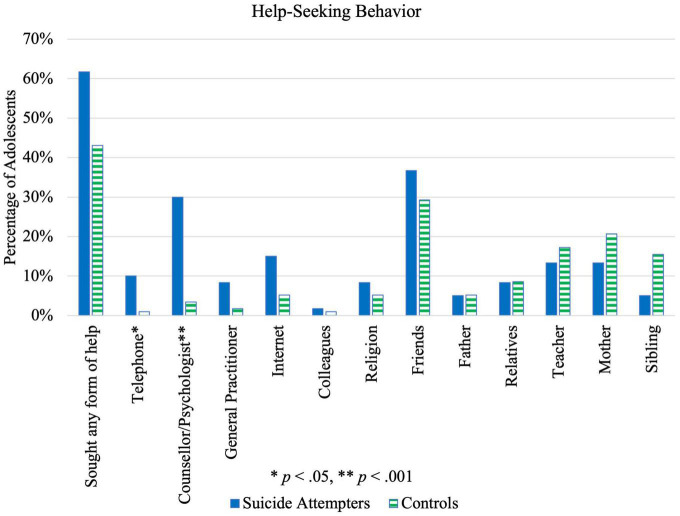
Illustration of differences in help-seeking behavior between suicide attempters and control group.

There were no statistically significant case-control differences with respect to whether any form of help was sought at all. Regarding specific help-seeking avenues, suicide attempters sought help from counselors/psychologists and telephone hotlines significantly more than controls, as seen in Table 7 (Figure 6). However, the proportion of attempters who sought help from telephone lines was small compared to other help-seeking avenues. For male adolescents, there were no statistically significant case-control differences in any help-seeking avenue, whereas for females, seeking help from counselors/psychologists (*p* = 0.001, OR = 10.14, 95% CI [2.14, 48.02]) and general practitioners (*p* = 0.026, OR = 12.71, 95% CI [0.68, 237.4]) was significantly higher among suicide attempters. There was no significant difference between males and females in terms of overall help-seeking behavior (*p* = 0.21).

**TABLE 7:** Comparison of help-seeking behavior between suicide attempters and control group.

Help-seeking avenues	Sample Size		*P*-value	Odds ratio	95% CI
	Suicide attempters^a^	Control^a^			
Sought any form of help	37 (61.7%)	25 (43.1%)	0.065	2.13	1.02 – 4.42
Telephone*	6 (10%)	0 (0%)	0.027	13.95^b^	0.77 – 253.61
Counsellor/Psychologist**	18 (30.0%)	2 (3.4%)	<0.001	12.00	2.64 – 54.58
General practitioner	5 (8.3%)	1 (1.7%)	0.207	5.18	0.59 – 45.78
Internet	9 (15%)	3 (5.2%)	0.126	3.24	0.83 – 12.62
Colleagues	1 (1.7%)	0 (0%)	1.000	2.95^b^	0.12 – 73.89
Religion	5 (8.3%)	3 (5.2%)	0.717	1.67	0.38 – 7.32
Friends	22 (36.7%)	17 (29.3%)	0.438	1.40	0.65 – 3.02
Father	3 (5%)	3 (5.2%)	1.000	0.97	0.19 – 4.99
Relatives	5 (8.3%)	5 (8.6%)	1.000	0.96	0.26 – 3.52
Teacher	8 (13.3%)	10 (17.2%)	0.615	0.74	0.27 – 2.03
Mother	8 (13.3%)	12 (20.7%)	0.333	0.59	0.22 – 1.57
Sibling	3 (5%)	9 (15.5%)	0.072	0.29	0.07 – 1.12

*P*-values calculated using Fishers’ Exact test. ^a^N(percentage); ^b^Modified Haldane-Anscombe correction applied due to presence of zero in contingency table, 0.5 was added to each cell; *p < 0.05, **p < 0.001.

Help-seeking behavior was expected to be more prevalent in cases than controls, especially from friends and family compared to healthcare professionals (42, 43). However, this was not observed in the present sample. While help-seeking from professionals may have resulted from higher school referrals for psychiatric help among adolescent suicide attempters, students have the choice to follow through or refuse these appointments. Future studies should examine the ways and rates of referral, and the factors that influence following through with these appointments.

Low levels of help sought from family members may indicate low perceived familial support, which aligns with adolescents’ perceptions of their parents’ parenting styles, as discussed above. Moreover, while help-seeking behavior may reflect social norms and stigma around mental health, it may also reflect the accessibility and structure of primary care and mental health services (42). Introduction of more community mental health services, in addition to campaigns to increase mental health literacy, could increase help-seeking behavior.

## Conclusion

The present study adopted a case-control approach to investigate the risk factors for suicide attempt in a multi-ethnic Asian population of 13-to-19-year-olds. All domains of life stress apart from emerging adult responsibility were higher among cases than controls, especially home life, peer pressure and romantic relationships. Suicide attempters tended to avoid new situations, be less adaptable to changes, have a negative outlook and irregular sleep-wake cycle. Additionally, they perceived their parents to be significantly more aggressive, neglecting, rejecting and cold, while parents’ perceptions of their own parenting were only significantly different in the domain of parental neglect. Cases were more likely to exhibit disorders of disruptive behavior, eating, mood, anxiety, symptoms of schizophrenia and experience of disturbing events. Significant differences were also found for 10 out of 12 Axis II disorders, particularly borderline, depressive, and avoidant personality disorder traits. No significant case-control differences were found regarding overall rates of help-seeking.

This study expands existing knowledge of adolescent suicide attempt risk factors to enhance the early identification of those at risk. Due to this study’s multi-ethnic demographic, findings may be generalizable to other Asian populations and could serve as a broad foundation for future studies examining suicidal behaviors in adolescents. The present findings implicate the means of identifying at-risk adolescents, through distinguishing those at greater suicide risk. This is particularly germane, since differentiating between distress, which is a normal response, and disturbance, which may reflect major underlying concerns, is challenging (40). Early identification must additionally work in tandem with early intervention strategies. Such strategies may draw from the findings presented in the present paper, such as the ways in which one may educate adolescents on stress management, empower them to seek help if needed, or expand their personal toolkit of coping strategies. However, broad-based school suicide awareness programs, suicide screening and lectures or focus groups on suicide might potentially be harmful or counterproductive (40). As such, targeted interventions based on early identification through understanding risk factors is key. Prevention strategies may include instilling greater hope and aspiration among adolescents through fostering positive peer relationships and financial literacy training, shaping resilience through interventions based on individuals’ temperament characteristics, and building a stronger home by educating and supporting parents. Campaigns to reduce stigma around mental health could additionally encourage greater help-seeking behavior.

### Limitations and future directions

A main limitation of this study is that retrospective self-report, which was this study’s primary avenue of data collection, may be subjected to inaccuracies of under- or over-reporting due to social desirability or recall bias, for caregivers and/or adolescents. To supplement the measure of adolescent temperament, collection of biomarkers may provide further insight and objective validation to the questionnaire data collected. This could include utilizing a biomarker-based temperament measure (44). Another key limitation of this study is its smaller sample size of 118 adolescents, with a higher representation of female participants. Gender bias should be accounted for. In addition, researchers should consider conducting a similar population profile study of adolescents who completed suicide through post-mortem retrospective assessments, to compare against those who survived a suicide attempt. This may inform a more holistic understanding of suicidal behavior. Future research may also consider a longitudinal approach to examining the relationship between these risk factors and adolescent suicidality. Finally, future studies should examine the ways in which the risk factors examined here interact with one another.

## Data availability statement

The datasets presented in this article are not readily available because of ethical reasons and privacy restrictions due to patient confidentiality and the sensitive nature of the data generated from this study. Anonymized data may be made available upon request by contacting the corresponding author, JW. Requests to access the datasets should be directed to JW, pcmwcmj@nus.edu.sg.

## Ethics statement

The studies involving human participants were reviewed and approved by National Healthcare Group Domain-Specific Review Board. Written informed consent to participate in this study was provided by the participants’ legal guardian/next of kin.

## Author contributions

JW was responsible for the design, development and overall conduct of the study. CC was responsible for data analysis and preparation of this manuscript. LS assisted in data analysis. NN and AL were responsible for conduct of the study and early data analysis. NY and LK supported the preparation of this manuscript. LF and CT were co-investigators for this study. All authors contributed to the article and approved the submitted version.
